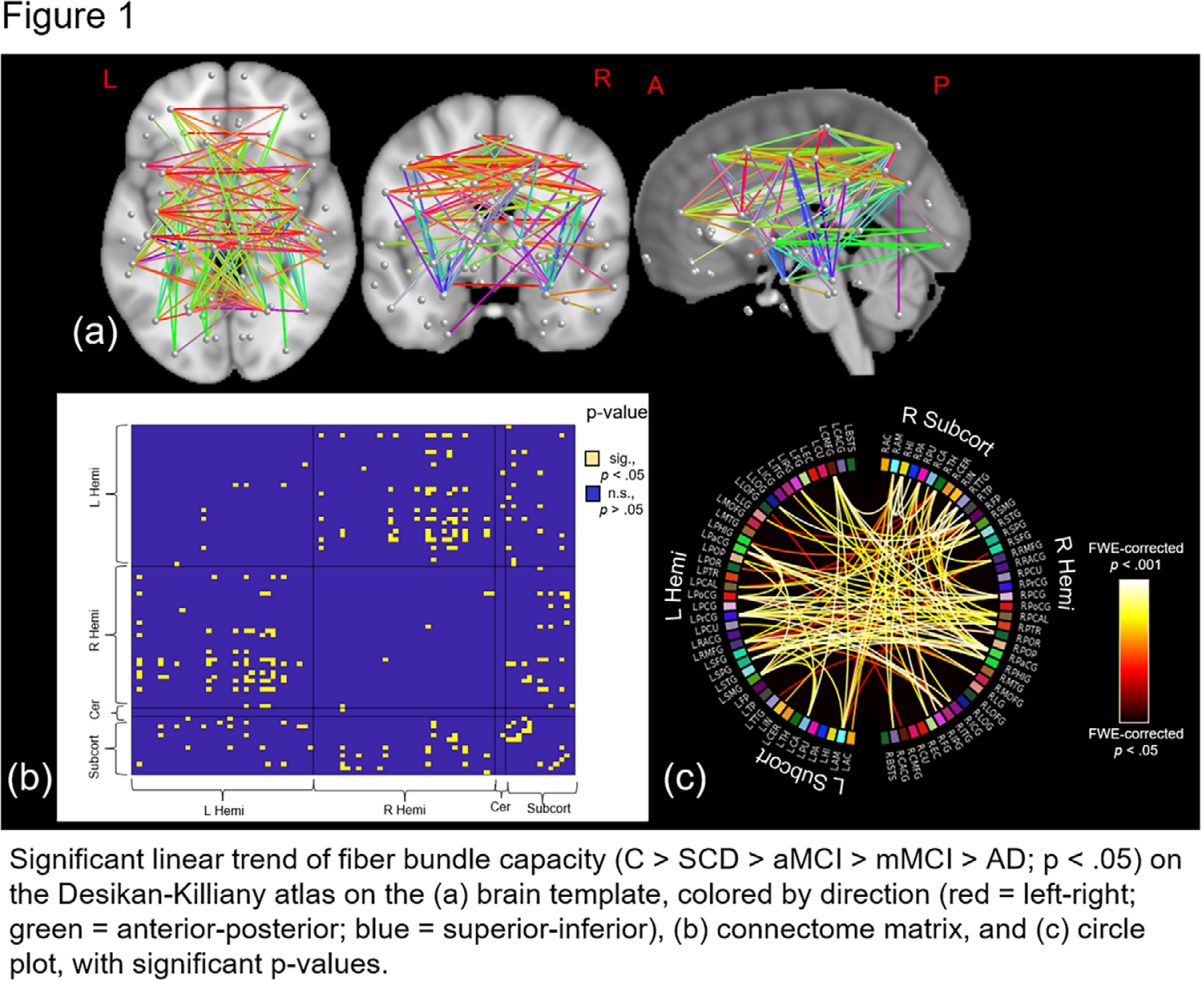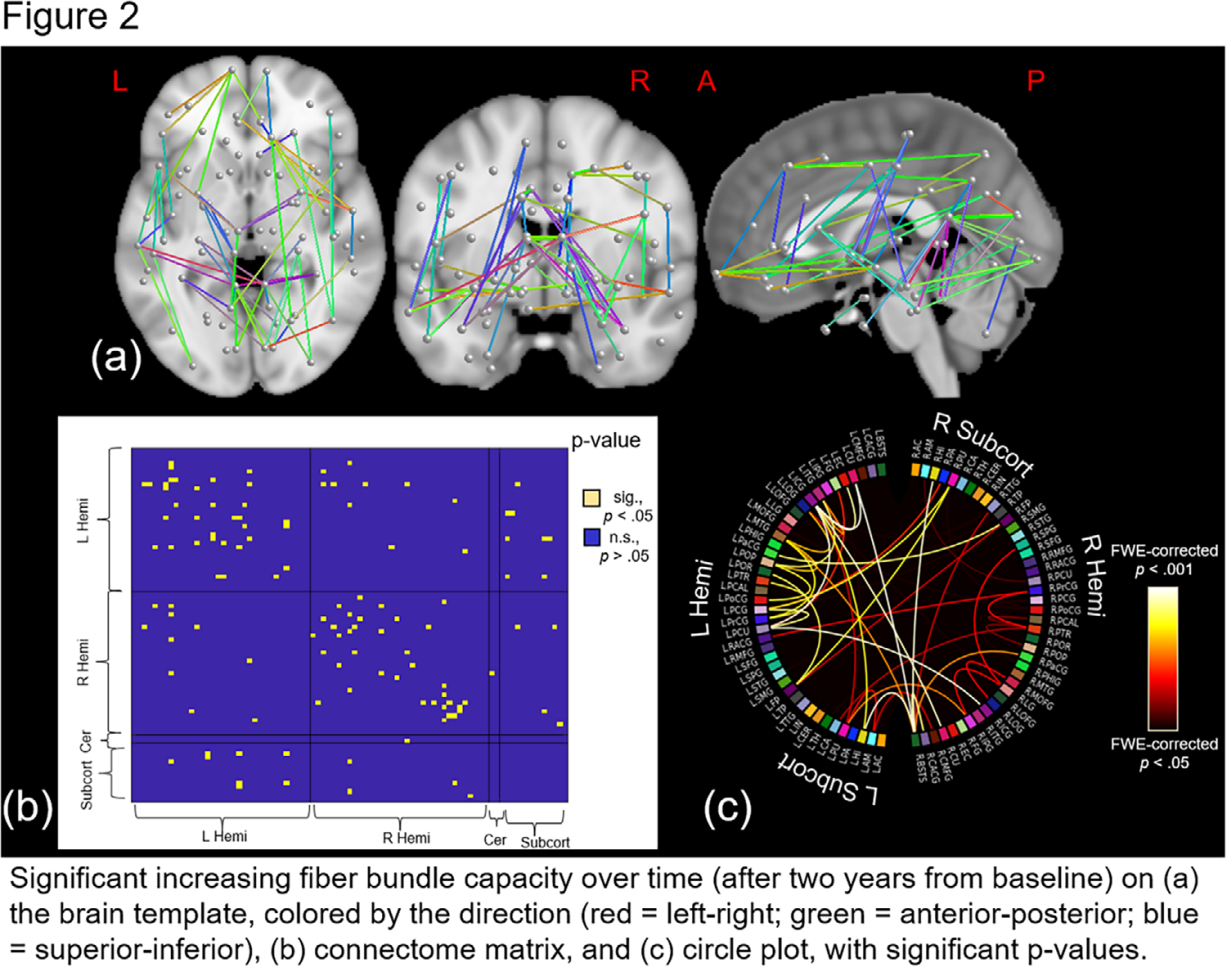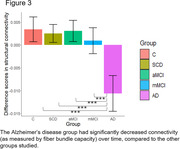# Investigation of structural connectivity in the whole brain and frontoparietal network in groups at risk of Alzheimer’s disease dementia

**DOI:** 10.1002/alz.094761

**Published:** 2025-01-09

**Authors:** Lenore T Tahara‐Eckl, Catherine A Morgan, Reece P Roberts, Flavio Dell'Acqua, Ian J Kirk, Tracy R Melzer, John C Dalrymple‐Alford, Tim J Anderson, Nicholas J Cutfield, Lynette J Tippett

**Affiliations:** ^1^ School of Psychology, The University of Auckland, Auckland New Zealand; ^2^ Centre for Brain Research, The University of Auckland, Auckland New Zealand; ^3^ Institute of Psychiatry, Psychology and Neuroscience, King’s College London, London United Kingdom; ^4^ New Zealand Brain Research Institute, Christchurch New Zealand; ^5^ School of Psychology, Speech and Hearing, University of Canterbury, Christchurch New Zealand; ^6^ Department of Medicine, University of Otago, Dunedin New Zealand

## Abstract

**Background:**

Brain connectivity patterns, measured with Magnetic Resonance Imaging (MRI), have been recently studied as a potential biomarker for Alzheimer’s disease dementia (AD). A ‘disconnected brain’ has been associated with greater cognitive impairment in pathological aging (Bennett & Madden, 2014), but it is unclear how this occurs in groups at risk of AD.

**Method:**

Participants (*n* = 227; aged 55+ years) from the Dementia Prevention Research Clinic, New Zealand, were classified as control (C; *n* = 35), subjective cognitive decline (SCD; *n* = 60), single‐domain amnestic mild cognitive impairment (aMCI; *n* = 54), multiple‐domain MCI (mMCI; *n* = 52), and AD (*n* = 26). Longitudinal changes (over two years) were investigated in a subset (total *n* = 119). Using diffusion MRI, we measured the white matter connections between brain regions with fiber bundle capacity (FBC), a proxy of white matter connection strength (Smith et al., 2022).

**Result:**

Cross‐sectionally, a significant linear trend of lower FBC was found across groups (C > SCD > aMCI > mMCI > AD; *p* < .05), mostly affecting interhemispheric connections (see Figure 1). Over time, FBC significantly decreased in only one connection between the left precentral and left parahippocampal region, but several intrahemispheric connections FBC connections increased over time (see Figure 2). A significant interaction (*F*(4, 114) = 10.60, *p* < .001, *η^2^* = 0.27) showed that the AD group had significantly decreased structural connectivity than all other groups over time (see Figure 3).

**Conclusion:**

We explored different threshold/processing strategies to ensure robust results. The preliminary results show the following: widespread disruption in structural connectivity was found cross‐sectionally in groups at higher risk of AD. Lower interhemispheric structural connectivity across groups may reflect degenerated commissural white matter tracts (e.g., corpus callosum) found in MCI and AD. The unexpected increasing intrahemispheric structural connectivity over time may suggest some structural reorganization, possibly in response to disrupted interhemispheric connections, or inflammation of the tracts. Alternatively, a general degeneration of commissural tracts may also explain an apparent increase of associative connectivity.

Bennett & Madden (2014). *Neuroscience*. https://doi.org/10.1016/j.neuroscience.2013.11.026

Smith et al. (2022). *Aperture Neuro*. https://doi.org/10.52294/apertureneuro.2022.2.neod9565